# Quinoline-2-sulfonamide

**DOI:** 10.1107/S160053681302062X

**Published:** 2013-07-31

**Authors:** Krzysztof Marciniec, Andrzej Maślankiewicz, Joachim Kusz, Maria Nowak

**Affiliations:** a Department of Organic Chemistry, The Medical University of Silesia, Jagiellońska 4, 41-200 Sosnowiec, Poland; b Institute of Physics, University of Silesia, Uniwersytecka 4, 40-007 Katowice, Poland

## Abstract

In the title compound, C_9_H_8_N_2_O_2_S, the sulfamoyl –NH_2_ group is involved in inter­molecular hydrogen bonding with the sulfonamide O and quinoline N atoms. In the crystal, mol­ecules are linked into dimers *via* pairs of N—H⋯N hydrogen bonds, forming an *R*_2_^2^(10) motif. The dimers are further assembled into chains parallel to the *b* axis through N—H⋯O hydrogen bonds, generating a *C*(4) motif. The crystal packing is additionally stabilized by inter­molecular C—H⋯O inter­actions. The crystal studied was a non-merohedral twin with a domain ratio of 0.938 (2):0.062 (2). Density functional theory (DFT) calculations, at the B3LYP/6–31 G(d,p) level of theory, were used to optimize the mol­ecular structure and to determine inter­action energies for the title compound. The resulting inter­action energy is ∼4.4 kcal mol^−1^ per bridge for the *C*(4) chain and ∼5.9 kcal mol^−1^ per bridge for the *R*_2_^2^(10) motif.

## Related literature

For the use of the quinoline­sulfamoyl unit in medicinal chemistry, see: Kim *et al.* (2005 ▶); Zajdel *et al.* (2012 ▶, 2013 ▶). For related structures, see: Marciniec *et al.* (2012 ▶). For the synthesis, see: Maślankiewicz *et al.* (2007 ▶). For hydrogen-bonding motifs in sufonamides, see: Adsmond & Grant (2001 ▶). For graph-set notation of hydrogen-bond motifs, see: Bernstein *et al.* (1995 ▶). For general hydrogen-bond rules, see: Donohue (1952 ▶); Etter (1990 ▶). For details of theoretical calculations, see: Frisch *et al.* (2009 ▶4); Parr & Yang (1989 ▶). The twin matrix was been determined with *ROTAX* (Cooper *et al.*, 2002 ▶).
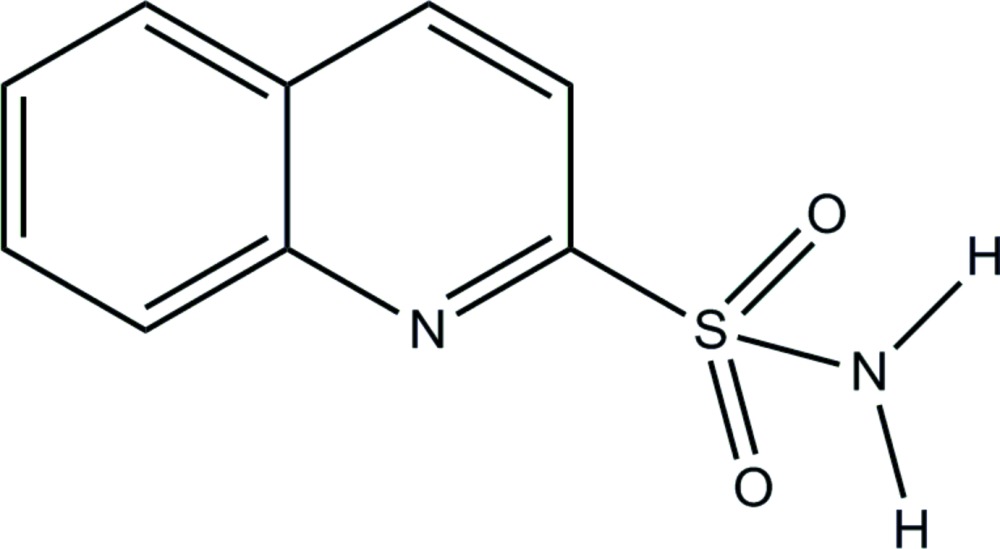


## Experimental

### 

#### Crystal data

C_9_H_8_N_2_O_2_S
*M* *_r_* = 208.23
Monoclinic, 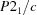

*a* = 8.5907 (1) Å
*b* = 5.1716 (1) Å
*c* = 20.0375 (3) Å
β = 94.230 (1)°
*V* = 887.79 (2) Å^3^
*Z* = 4
Mo *K*α radiation
μ = 0.34 mm^−1^
*T* = 100 K
0.27 × 0.23 × 0.05 mm

#### Data collection

Agilent SuperNova diffractometer with an Atlas detector
Absorption correction: multi-scan (*CrysAlis PRO*; Agilent, 2011 ▶) *T*_min_ = 0.919, *T*_max_ = 1.000
27311 measured reflections
1552 independent reflections
1530 reflections with *I* > 2σ(*I*)
*R*_int_ = 0.024

#### Refinement

*R*[*F* ^2^ > 2σ(*F* ^2^)] = 0.030
*wR*(*F* ^2^) = 0.072
*S* = 1.12
1552 reflections
160 parameters
All H-atom parameters refined
Δρ_max_ = 0.35 e Å^−3^
Δρ_min_ = −0.32 e Å^−3^

### 

Data collection: *CrysAlis PRO* (Agilent, 2011 ▶); cell refinement: *CrysAlis PRO*; data reduction: *CrysAlis PRO*; program(s) used to solve structure: *SHELXS97* (Sheldrick, 2008 ▶); program(s) used to refine structure: *SHELXL97* (Sheldrick, 2008 ▶) and *WinGX* (Farrugia, 2012 ▶); molecular graphics: *ORTEP-3 for Windows* (Farrugia, 2012 ▶) and *Mercury* (Macrae *et al.*, 2006 ▶); software used to prepare material for publication: *publCIF* (Westrip, 2010 ▶).

## Supplementary Material

Crystal structure: contains datablock(s) I, New_Global_Publ_Block. DOI: 10.1107/S160053681302062X/gk2577sup1.cif

Structure factors: contains datablock(s) I. DOI: 10.1107/S160053681302062X/gk2577Isup2.hkl

Supplementary material file. DOI: 10.1107/S160053681302062X/gk2577Isup3.cml

Additional supplementary materials:  crystallographic information; 3D view; checkCIF report

## Figures and Tables

**Table 1:** Hydrogen-bond geometry (Å, °)

*D*—H⋯*A*	*D*—H	H⋯*A*	*D*⋯*A*	*D*—H⋯*A*
N2—H2*N*2⋯O1^i^	0.84 (3)	2.09 (3)	2.922 (2)	171 (2)
N2—H1*N*2⋯N1^ii^	0.80 (3)	2.18 (3)	2.962 (2)	165 (2)
C6—H6⋯O1^iii^	0.93 (2)	2.66 (2)	3.431 (2)	141.5 (18)

Symmetry codes: (i) 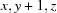; (ii) 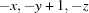; (iii) 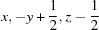.

**Fig. 1.**
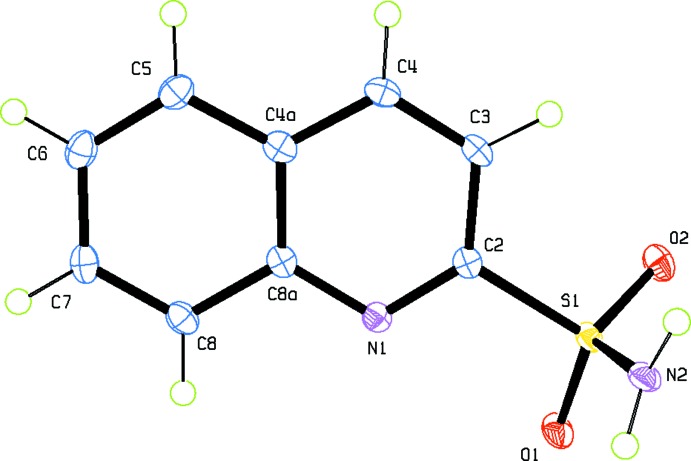
The molecular structure of the title compound with the atom labeling and displacement ellipsoids drawn at the 50% probability level.

**Fig. 2.**
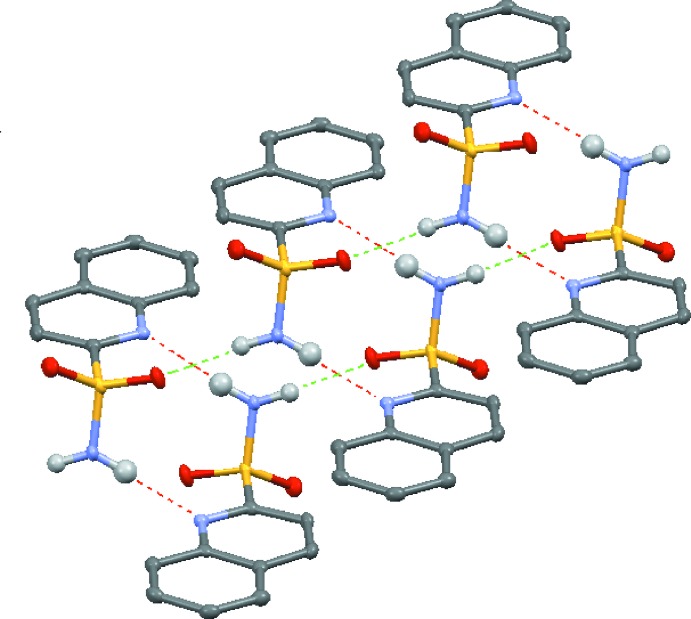
Intermolecular N-H···N and N-H···O hydrogen bonds (dashed lines) in the title compound

**Fig. 3.**
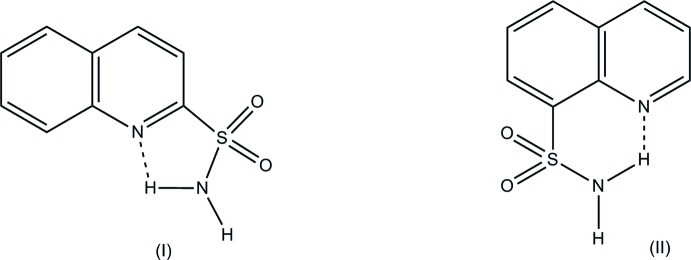
Intramolecular hydrogen bond in 2-quinolinesulfonamide (I) (calculated) and 8-quinolinesulfonamide (II) (experimentally determined).

## supplementary crystallographic information

### Comment

Compounds containing quinolinesulfamoyl moiety have received considerable attention in recent years due to their diverse pharmacological properties including antidepressant (Zajdel *et al.*, 2012; Zajdel *et al.*, 2013) or anticancer activity (Kim *et al.*, 2005).

From a structural point of view, sulfonamides are interesting because of their tendency to form different hydrogen-bond patterns in the solid state. Studies have shown that primary hydrogen-bond connectivity is somewhat predictable. Two general hydrogen-bond rules based on empirical observations of hundreds of crystal structures over the years have been developed that summarize this predictability (Adsmond & Grant, 2001). The first of these rules states "all good donors and acceptors are used in hydrogen bonding" (Donohue, 1952). The second hydrogen-bond rule for crystal structures of small organic molecules states that, after formation of intramolecular hydrogen bonds, the best hydrogen bond donor will bond to the best hydrogen-bond acceptor present (Etter, 1990). The molecule of quinolinesulfonamide has two good hydrogen bond donors (the sulfonamido H atoms) and three the best hydrogen bond acceptors (the sulfonamido O atoms and quinoline nitrogen atom). We have previously reported the X-ray crystal structure of quinoline-8-sulfonamide (Marciniec *et al.*, 2012). The obtained results show, according to the second hydrogen-bond rule, that the sulfamoyl NH_2_ group is involved in intramolecular N—H···N hydrogen bond resulting in the graph-set motif of *S*(6) (Bernstein *et al.* 1995). After formation of intramolecular hydrogen bond, the sulfamoyl NH_2_ group is involved in intermolecular N—H···O hydrogen bond resulting in the graph-set motif of *R*_2_^2^(8). The key feature of the molecular structure of 2-quinolinesulfonamide (I) (Fig.1) is the N1—C2—S1—N2 torsion angle of -95.88 (14)°. The geometry of the sulfonamide group does not allow for intramolecular hydrogen-bond ring formation. In the crystal structure, the molecules form dimers through N2—H1···N1 intermolecular hydrogen bonds [graph set *R*_2_^2^(10)] which are extended into one-dimensional chains [graph set *C*(4)] along the *b* axis, through the sulfonamide N2—H2···O1 hydrogen bonds (Table 1; Fig. 2). Thus the first-level graph set is N_1_= *C*(4) *R*_2_^2^(10).

The second-level graph-set notation (for combinations of two hydrogen bonds: N2—H2···O1 and N2—H1···N1) was determined to be as follows N_2_= *R*_4_^4^(14)*C*_4_^4^(16) *R*_4_^4^(16) *R*_4_^4^(18) *R*_6_^6^(22) *R*_6_^6^(24) *R*_6_^6^(26).

The crystal packing is further stabilized by week intermolecular hydrogen bonds C3—H3···O2 and C4—H4···O2 which generate *R*_2_^1^(5) ring and C6—H6···O1 hydrogen bond which generates *D* motif.

The molecular structure of (I) in the gas phase was optimized by the density-functional theory at the B3LYP/6–31 G(d,p) level, using the computer program *GAUSSIAN*09 (Parr & Yang, 1989; Frisch *et al.*, 2009). Calculations were performed using the X-ray coordinates as the input structure. The calculated geometry of the sulfonamide group allows for intramolecular hydrogen-bond five-membered ring formation (H1···N1 = 2.618 Å; N2—H1···N1 = 100.3°; N1—C2—S1—N2 = -35.2°) (Fig. 3). In the crystal structure of quinoline-2-sulfonamide (I) an intermolecular hydrogen bond is more difficult to break than comparable intramolecular hydrogen bond formed between the the sulfamoyl NH_2_ group and endocyclic nitrogen

The minimum-energy structure of sulfonamides in the gas phase might not be identical to that observed in the solid state. Intermolecular interactions, and hydrogen bonding in particular, might strongly influence the conformations adopted in the solid state, whereas intramolacular interactions and and hydrogen bonding will dominate the gas-phase conformation.

The interaction energies for 2-quinolinesulfonamide (I) were investigated by adding successive units, in their crystallographic positions, once for the chain [graph set *C*(4)], once for the centrosymmetric dimer [graph set *R*_2_^2^(10)]. These energies were then compared with the energy for the same number of independent asymmetric units to determine the stabilization energy for the interaction between units. The results are presented in table 2.

The energy difference as a function of the number of bridging interactions is nearly linear and independent of choice of generated cavity. The resulting interaction energy is ~4.4 kcal/mol/bridge for *C*(4) chain and ~5.9 kcal/mol/bridge for *R*_2_^2^(10) dimer. The small increase in stabilization energy per unit as the number of formula units suggests that there are negligible contributions from extended molecular orbitals, supporting the original assumption that the significant interactions result from hydrogen bonding contributions.

### Experimental

The title compound was prepared by the reaction of hydrochloric acid solution of quinoline-2-sulfochloride with an excess ammonia at -10 °C according to the procedure reported by Maślankiewicz *et al.* (2007). Single crystals of the title compound suitable for X-ray structure determination were obtained by recrystallization from an ethanolic solution.

### Refinement

The hydrogen atoms participating in hydrogen bonding were located in a difference Fourier map and freely refined. The twin matrix, 1 0 0 0 - 1 0 - 0.344 0 - 1, has been determined with the ROTAX program (Cooper *et al.*, 2002). For the further refinement, the reflection data file in HKLF 5 format was prepared using "Make HKLF5" function of the *WinGX* program (Farrugia, 2012).

### Crystal data

**Table:**

C_9_H_8_N_2_O_2_S	*F*(000) = 432
*M**_r_* = 208.23	*D*_x_ = 1.558 Mg m^−^^3^
Monoclinic, *P*2_1_/*c*	Melting point: 441.2 K
Hall symbol: -P 2ybc	Mo *K*α radiation, λ = 0.71073 Å
*a* = 8.5907 (1) Å	Cell parameters from 14635 reflections
*b* = 5.1716 (1) Å	θ = 2.0–37.3°
*c* = 20.0375 (3) Å	µ = 0.34 mm^−^^1^
β = 94.230 (1)°	*T* = 100 K
*V* = 887.79 (2) Å^3^	Plate, colorless
*Z* = 4	0.27 × 0.23 × 0.05 mm

### Data collection

**Table:**

Agilent SuperNova diffractometer with an Atlas detector	1552 independent reflections
Radiation source: SuperNova (Mo) X-ray Source	1530 reflections with *I* > 2σ(*I*)
Mirror monochromator	*R*_int_ = 0.024
Detector resolution: 10.4498 pixels mm^-1^	θ_max_ = 25.1°, θ_min_ = 2.0°
ω scans	*h* = −10→10
Absorption correction: multi-scan (*CrysAlis PRO*; Agilent, 2011)	*k* = −6→6
*T*_min_ = 0.919, *T*_max_ = 1.000	*l* = −23→23
27311 measured reflections	

### Refinement

**Table:**

Refinement on *F*^2^	Primary atom site location: structure-invariant direct methods
Least-squares matrix: full	Secondary atom site location: difference Fourier map
*R*[*F*^2^ > 2σ(*F*^2^)] = 0.030	Hydrogen site location: inferred from neighbouring sites
*wR*(*F*^2^) = 0.072	All H-atom parameters refined
*S* = 1.12	*w* = 1/[σ^2^(*F*_o_^2^) + (0.0262*P*)^2^ + 0.9337*P*] where *P* = (*F*_o_^2^ + 2*F*_c_^2^)/3
1552 reflections	(Δ/σ)_max_ < 0.001
160 parameters	Δρ_max_ = 0.35 e Å^−^^3^
0 restraints	Δρ_min_ = −0.32 e Å^−^^3^

### Special details

**Table:**

Geometry. All e.s.d.'s (except the e.s.d. in the dihedral angle between two l.s. planes) are estimated using the full covariance matrix. The cell e.s.d.'s are taken into account individually in the estimation of e.s.d.'s in distances, angles and torsion angles; correlations between e.s.d.'s in cell parameters are only used when they are defined by crystal symmetry. An approximate (isotropic) treatment of cell e.s.d.'s is used for estimating e.s.d.'s involving l.s. planes.
Refinement. Refinement of *F*^2^ against ALL reflections. The weighted *R*-factor *wR* and goodness of fit *S* are based on *F*^2^, conventional *R*-factors *R* are based on *F*, with *F* set to zero for negative *F*^2^. The threshold expression of *F*^2^ > σ(*F*^2^) is used only for calculating *R*-factors(gt) *etc*. and is not relevant to the choice of reflections for refinement. *R*-factors based on *F*^2^ are statistically about twice as large as those based on *F*, and *R*- factors based on ALL data will be even larger.

### Fractional atomic coordinates and isotropic or equivalent isotropic displacement parameters (Å^2^)

**Table:**

	*x*	*y*	*z*	*U*_iso_*/*U*_eq_	
S1	0.24131 (5)	0.61370 (8)	0.04453 (2)	0.01307 (14)	
O1	0.18557 (15)	0.3614 (2)	0.06117 (6)	0.0193 (3)	
O2	0.38294 (14)	0.7120 (3)	0.07708 (6)	0.0188 (3)	
N1	0.19733 (16)	0.4337 (3)	−0.07942 (7)	0.0132 (3)	
N2	0.10504 (19)	0.8125 (3)	0.05580 (8)	0.0164 (3)	
C2	0.26951 (19)	0.6149 (3)	−0.04339 (8)	0.0129 (4)	
C3	0.3627 (2)	0.8112 (4)	−0.06810 (9)	0.0153 (4)	
C4	0.3835 (2)	0.8125 (4)	−0.13486 (9)	0.0156 (4)	
C4A	0.31105 (19)	0.6202 (4)	−0.17656 (9)	0.0147 (4)	
C5	0.3263 (2)	0.6090 (4)	−0.24669 (9)	0.0169 (4)	
C6	0.2521 (2)	0.4209 (4)	−0.28441 (9)	0.0180 (4)	
C7	0.1578 (2)	0.2367 (4)	−0.25488 (9)	0.0175 (4)	
C8	0.1389 (2)	0.2425 (4)	−0.18758 (9)	0.0159 (4)	
C8A	0.21642 (19)	0.4342 (3)	−0.14675 (8)	0.0129 (4)	
H2N2	0.126 (3)	0.971 (5)	0.0528 (11)	0.027 (6)*	
H1N2	0.019 (3)	0.751 (5)	0.0549 (12)	0.035 (7)*	
H3	0.409 (2)	0.940 (4)	−0.0389 (11)	0.020 (5)*	
H4	0.444 (2)	0.945 (4)	−0.1538 (10)	0.021 (5)*	
H5	0.391 (2)	0.744 (4)	−0.2674 (9)	0.012 (5)*	
H6	0.265 (3)	0.415 (4)	−0.3299 (12)	0.026 (6)*	
H7	0.107 (2)	0.103 (4)	−0.2823 (11)	0.023 (6)*	
H8	0.073 (2)	0.117 (4)	−0.1661 (11)	0.023 (6)*	

### Atomic displacement parameters (Å^2^)

**Table:**

	*U*^11^	*U*^22^	*U*^33^	*U*^12^	*U*^13^	*U*^23^
S1	0.0168 (2)	0.0096 (2)	0.0124 (2)	−0.00169 (17)	−0.00137 (16)	−0.00008 (16)
O1	0.0290 (7)	0.0112 (6)	0.0172 (6)	−0.0037 (6)	−0.0005 (5)	0.0013 (5)
O2	0.0188 (6)	0.0195 (7)	0.0172 (6)	−0.0019 (6)	−0.0046 (5)	−0.0012 (5)
N1	0.0128 (7)	0.0115 (7)	0.0152 (7)	0.0013 (6)	−0.0002 (6)	−0.0011 (6)
N2	0.0166 (8)	0.0106 (8)	0.0223 (8)	−0.0043 (7)	0.0030 (6)	−0.0020 (6)
C2	0.0114 (8)	0.0123 (9)	0.0146 (8)	0.0028 (7)	−0.0011 (6)	−0.0010 (7)
C3	0.0142 (8)	0.0135 (9)	0.0176 (9)	−0.0015 (7)	−0.0023 (7)	−0.0015 (7)
C4	0.0119 (8)	0.0144 (9)	0.0203 (9)	−0.0007 (7)	0.0007 (7)	0.0014 (7)
C4A	0.0116 (8)	0.0149 (9)	0.0176 (9)	0.0023 (7)	0.0002 (7)	0.0005 (7)
C5	0.0153 (8)	0.0187 (9)	0.0169 (9)	0.0017 (8)	0.0029 (7)	0.0015 (7)
C6	0.0164 (9)	0.0225 (10)	0.0149 (9)	0.0048 (8)	0.0006 (7)	−0.0015 (8)
C7	0.0167 (9)	0.0177 (9)	0.0173 (9)	0.0031 (8)	−0.0032 (7)	−0.0046 (7)
C8	0.0138 (8)	0.0146 (9)	0.0192 (9)	0.0006 (7)	−0.0005 (7)	−0.0010 (7)
C8A	0.0112 (8)	0.0116 (8)	0.0157 (9)	0.0033 (7)	−0.0010 (6)	−0.0004 (7)

### Geometric parameters (Å, º)

**Table:**

S1—O2	1.4308 (13)	C4—H4	0.95 (2)
S1—O1	1.4376 (13)	C4A—C8A	1.419 (2)
S1—N2	1.5866 (16)	C4A—C5	1.422 (2)
S1—C2	1.7959 (18)	C5—C6	1.361 (3)
N1—C2	1.311 (2)	C5—H5	1.00 (2)
N1—C8A	1.371 (2)	C6—C7	1.409 (3)
N2—H2N2	0.84 (3)	C6—H6	0.93 (2)
N2—H1N2	0.80 (3)	C7—C8	1.370 (3)
C2—C3	1.406 (3)	C7—H7	0.97 (2)
C3—C4	1.363 (3)	C8—C8A	1.419 (2)
C3—H3	0.96 (2)	C8—H8	0.98 (2)
C4—C4A	1.414 (3)		
			
O2—S1—O1	120.23 (8)	C4—C4A—C8A	117.98 (16)
O2—S1—N2	108.44 (8)	C4—C4A—C5	122.96 (17)
O1—S1—N2	107.06 (9)	C8A—C4A—C5	119.05 (16)
O2—S1—C2	105.91 (8)	C6—C5—C4A	120.28 (17)
O1—S1—C2	107.59 (8)	C6—C5—H5	121.3 (11)
N2—S1—C2	106.96 (8)	C4A—C5—H5	118.4 (11)
C2—N1—C8A	117.05 (15)	C5—C6—C7	120.69 (17)
S1—N2—H2N2	117.0 (16)	C5—C6—H6	118.9 (14)
S1—N2—H1N2	115.3 (19)	C7—C6—H6	120.4 (14)
H2N2—N2—H1N2	126 (3)	C8—C7—C6	120.79 (17)
N1—C2—C3	125.52 (17)	C8—C7—H7	119.6 (13)
N1—C2—S1	116.44 (13)	C6—C7—H7	119.6 (13)
C3—C2—S1	118.02 (13)	C7—C8—C8A	119.87 (17)
C4—C3—C2	117.95 (17)	C7—C8—H8	122.0 (13)
C4—C3—H3	121.2 (13)	C8A—C8—H8	118.1 (13)
C2—C3—H3	120.9 (13)	N1—C8A—C8	118.75 (16)
C3—C4—C4A	119.54 (17)	N1—C8A—C4A	121.94 (16)
C3—C4—H4	120.5 (13)	C8—C8A—C4A	119.31 (16)
C4A—C4—H4	119.9 (13)		
			
C8A—N1—C2—C3	1.0 (3)	C4—C4A—C5—C6	−179.17 (17)
C8A—N1—C2—S1	179.28 (12)	C8A—C4A—C5—C6	−0.5 (3)
O2—S1—C2—N1	148.59 (13)	C4A—C5—C6—C7	0.7 (3)
O1—S1—C2—N1	18.84 (15)	C5—C6—C7—C8	−0.1 (3)
N2—S1—C2—N1	−95.88 (14)	C6—C7—C8—C8A	−0.7 (3)
O2—S1—C2—C3	−32.97 (16)	C2—N1—C8A—C8	−179.41 (15)
O1—S1—C2—C3	−162.72 (13)	C2—N1—C8A—C4A	0.6 (2)
N2—S1—C2—C3	82.55 (15)	C7—C8—C8A—N1	−179.13 (16)
N1—C2—C3—C4	−1.5 (3)	C7—C8—C8A—C4A	0.9 (3)
S1—C2—C3—C4	−179.75 (13)	C4—C4A—C8A—N1	−1.6 (2)
C2—C3—C4—C4A	0.4 (3)	C5—C4A—C8A—N1	179.74 (15)
C3—C4—C4A—C8A	1.0 (3)	C4—C4A—C8A—C8	178.43 (16)
C3—C4—C4A—C5	179.67 (17)	C5—C4A—C8A—C8	−0.3 (2)

### Hydrogen-bond geometry (Å, º)

**Table:**

*D*—H···*A*	*D*—H	H···*A*	*D*···*A*	*D*—H···*A*
N2—H2*N*2···O1^i^	0.84 (3)	2.09 (3)	2.922 (2)	171 (2)
N2—H1*N*2···N1^ii^	0.80 (3)	2.18 (3)	2.962 (2)	165 (2)
C3—H3···O2^iii^	0.96 (2)	2.68 (2)	3.308 (2)	123.5 (16)
C4—H4···O2^iii^	0.95 (2)	2.72 (2)	3.327 (2)	122.3 (15)
C6—H6···O1^iv^	0.93 (2)	2.66 (2)	3.431 (2)	141.5 (18)

Symmetry codes: (i) *x*, *y*+1, *z*; (ii) −*x*, −*y*+1, −*z*; (iii) −*x*+1, −*y*+2, −*z*; (iv) *x*, −*y*+1/2, *z*−1/2.

### Table 2. Calculation of stabilization energies for quinoline-2-sulfonamide (kcal mol^-1^)

**Table:**

	B3LYP/6-31G(d,p)	
	Energy	ΔE
Asymmetric unit	-631075.7	
2 units N_1_ = *C*(4)	-1262155.9	-4.4
2 units N_1_ = *R*_2_^2^(10)	-1262163.3	-11.8
3 units N_1_ = *C*(4)*R*_2_^2^(10)	-1893243.7	-16.6
4 units N_2_ = *R*_4_^4^(14)	-2524335.0	-32.2

## References

[bb1] Adsmond, D. A. & Grant, D. J. W. (2001). *J. Pharm. Sci.* **90**, 2058–2077. 10.1002/jps.1157 11745765

[bb2] Agilent (2011). *CrysAlis PRO* Agilent Technologies Ltd, Yarnton, England.

[bb3] Bernstein, J., Davis, R. E., Shimoni, L. & Chang, N.-L. (1995). *Angew. Chem. Int. Ed. Engl.* **34**, 1555–1573.

[bb4] Cooper, R. I., Gould, R. O., Parsons, S. & Watkin, D. J. (2002). *J. Appl. Cryst.* **35**, 168–174.

[bb5] Donohue, J. (1952). *J. Phys. Chem.* **56**, 502–510.

[bb6] Etter, M. C. (1990). *Acc. Chem. Res.* **23**, 120–126.

[bb7] Farrugia, L. J. (2012). *J. Appl. Cryst.* **45**, 849–854.

[bb8] Frisch, M. J., *et al.* (2009). *GAUSSIAN09* Gaussian, Inc., Wallingford, CT, USA.

[bb9] Kim, Y.-H., Shin, K.-J., Lee, T. G., Kim, E., Lee, M.-S., Ryu, S. H. & Suh, P.-G. (2005). *Biochem. Pharmacol.* **69**, 1333–1341. 10.1016/j.bcp.2004.12.019 15826604

[bb10] Macrae, C. F., Edgington, P. R., McCabe, P., Pidcock, E., Shields, G. P., Taylor, R., Towler, M. & van de Streek, J. (2006). *J. Appl. Cryst.* **39**, 453–457.

[bb11] Marciniec, K., Maślankiewicz, A., Nowak, M. & Kusz, J. (2012). *Acta Cryst.* E**68**, o2826. 10.1107/S1600536812036963 PMC3470188 23125632

[bb12] Maślankiewicz, A., Marciniec, K., Pawłowski, M. & Zajdel, P. (2007). *Heterocycles*, **71**, 1975–1990.

[bb13] Parr, R. G. & Yang, W. (1989). In *Density Functional Theory of Atoms and Molecules* New York: Oxford University Press Inc.

[bb14] Sheldrick, G. M. (2008). *Acta Cryst.* A**64**, 112–122. 10.1107/S0108767307043930 18156677

[bb15] Westrip, S. P. (2010). *J. Appl. Cryst.* **43**, 920–925.

[bb16] Zajdel, P., Marciniec, K., Grychowska, K., Maślankiewicz, A., Satała, G., Duszyńska, B., Siwek, A., Nowak, G., Partyka, A., Wróbel, D., Jastrzębska-Więsek, M., Bojarski, A. J., Wesołowska, A. & Pawłowski, M. (2013). *Eur. J. Med. Chem.* **60**, 42–50. 10.1016/j.ejmech.2012.11.042 23279866

[bb17] Zajdel, P., Marciniec, K., Maślankiewicz, A., Satała, G., Duszyńska, B., Bojarski, A. J., Partyka, A., Jastrzębska-Więsek, M., Wróbel, D., Wesołowska, A. & Pawłowski, M. (2012). *Bioorg. Med. Chem.* **20**, 1545–1556. 10.1016/j.bmc.2011.12.039 22277589

